# Acylpeptide Hydrolase Inhibition as Targeted Strategy to Induce Proteasomal Down-Regulation

**DOI:** 10.1371/journal.pone.0025888

**Published:** 2011-10-10

**Authors:** Gianna Palmieri, Paolo Bergamo, Alberto Luini, Menotti Ruvo, Marta Gogliettino, Emma Langella, Michele Saviano, Ramanath N. Hegde, Annamaria Sandomenico, Mose Rossi

**Affiliations:** 1 Institute of Protein Biochemistry, National Research Council (CNR-IBP), Napoli, Italy; 2 Institute of Food Sciences, National Research Council (CNR-ISA), Avellino, Italy; 3 Institute of Biostructure and Bioimaging, National Research Council (CNR-IBB), Napoli, Italy; 4 Institute of Crystallography, National Council of Research of Italy (CNR-IC), Bari, Italy; 5 Telethon Institute of Genetics and Medicine (TIGEM), Napoli, Italy; Wayne State University, United States of America

## Abstract

Acylpeptide hydrolase (APEH), one of the four members of the prolyl oligopeptidase class, catalyses the removal of N-acylated amino acids from acetylated peptides and it has been postulated to play a key role in protein degradation machinery. Disruption of protein turnover has been established as an effective strategy to down-regulate the ubiquitin-proteasome system (UPS) and as a promising approach in anticancer therapy.

Here, we illustrate a new pathway modulating UPS and proteasome activity through inhibition of APEH. To find novel molecules able to down-regulate APEH activity, we screened a set of synthetic peptides, reproducing the reactive-site loop of a known archaeal inhibitor of APEH (SsCEI), and the conjugated linoleic acid (CLA) isomers. A 12-mer SsCEI peptide and the trans10-cis12 isomer of CLA, were identified as specific APEH inhibitors and their effects on cell-based assays were paralleled by a dose-dependent reduction of proteasome activity and the activation of the pro-apoptotic caspase cascade. Moreover, cell treatment with the individual compounds increased the cytoplasm levels of several classic hallmarks of proteasome inhibition, such as NFkappaB, p21, and misfolded or polyubiquitinylated proteins, and additive effects were observed in cells exposed to a combination of both inhibitors without any cytotoxicity. Remarkably, transfection of human bronchial epithelial cells with APEH siRNA, promoted a marked accumulation of a mutant of the cystic fibrosis transmembrane conductance regulator (CFTR), herein used as a model of misfolded protein typically degraded by UPS. Finally, molecular modeling studies, to gain insights into the APEH inhibition by the trans10-cis12 CLA isomer, were performed.

Our study supports a previously unrecognized role of APEH as a negative effector of proteasome activity by an unknown mechanism and opens new perspectives for the development of strategies aimed at modulation of cancer progression.

## Introduction

In all living cells, proteolysis is essential in the control of many basic processes, including protein quality control, cell-cycle progression, signal transduction, apoptosis, and gene expression. One of the major players in the regulation of intracellular proteolysis is the ubiquitin-proteasome system (UPS) [1]. This is a complex enzymatic machine that primarily contributes to the cytoplasmic turnover of a vast majority of proteins in mammalian cells and it is tightly controlled by a number of endogenous regulators. Due to the multiple roles of UPS, it is essential in eukaryotes and its dysfunction can have deleterious effects in cells and for the organism as a whole. UPS dysregulation has been implicated in a number of pathologies such as autoimmune, neurodegenerative diseases and viral infections, and it is considered a novel therapeutic target for tackling tumoral diseases [2]–[6]. Indeed, protein homeostasis is critically involved in cancer cell survival thus, targeting the balance between the production and destruction of proteins mediating cell proliferation, has become a major focus in cancer research. Accordingly, over the past decade, several studies have been focused on the development of specific proteasome inhibitors (PIs), which have relevant anticancer effects and particularly on those involving in the repression of nuclear factor-κ (NF-κ B) signalling, and in the promotion of apoptosis in transformant cells [7], [8].

The first evidence of the pro-apoptotic activity of PIs was shown in U937 human monoblast cells [9]. In 2003, the PI bortezomib (Velcade® or PS341) has been approved by the Food and Drug Administration for the treatment of multiple myeloma, which confirmed the efficacy of PIs in blocking cancer progression. However, like other PIs, bortezomib has several relevant adverse events [10], [11] and, at present, increasing research efforts are aimed at reducing these negative side-effects through the use of inhibitors with reversible and time-limited binding activity and increased bioavailability. Recent studies have suggested that some fatty acids are able to disrupt the chymotrypsin (CT)-like proteasome activity. Among these, the two major isomers of conjugated linoleic acid (CLA), cis9-trans11 CLA (c_9_t_11_-CLA) and trans10-cis12 CLA (t_10_c_12_-CLA), have shown pro-apoptotic activities in a number of cancer cell lines [12] and strong anticancer effects in numerous animal models [13]. Interestingly, although the mechanisms are yet poorly understood, their ability to inhibit the proteasome activity *in vitro* [14] suggests that this complex enzyme could be their ultimate target.

Acylpeptide hydrolase (APEH; also known as acylaminoacyl peptidase or oxidised protein hydrolase) is one of the four members of the prolyl oligopeptidase class (POP, clan SC, family S9); it catalyses the removal of N-acylated amino acids from acetylated peptides and has been recently recognised as having a role in the coordinated protein-degradation machinery in Cos-7 cells [15], and in the modulation of cancer progression [16].

On this background, we have investigated the molecular mechanisms that underlie the interrelationship between APEH and the proteasome, and their eventual regulation by natural or synthetic compounds including peptides reproducing the reactive site loop (RSL) of an archaeal APEH inhibitor (SsCEI, *Sulfolobus solfataricus* chymotrypsin-elastase inhibitor) [17], and the c_9_t_11_-CLA and t_10_c_12_-CLA isomers. Two molecules that selectively inhibit APEH and induce, in parallel, a down-regulation of proteasome activity have been identified. Moreover, a direct correlation between APEH inhibition and proteasome down-regulation has been established using a specific APEH siRNA probe. A molecular docking analysis has been also carried out to predict the CLA-enzyme binding sites. Therefore, this study shows that proteasome functions can be upstream regulated by APEH, and that inhibition of APEH activity appears to be an important event in controlling the proteasome dysfunction associated with pathological conditions, opening new important and challenging perspectives for the development of novel strategies in cancer therapy.

## Results and Discussion

### Peptide design and characterisation

The recent identification and characterisation of an endogenous inhibitor protein of APEH in S. solfataricus (SsCEI) [17], [18], was the starting point for the present study and the S. solfataricus APEH (APEHSs) was used, in a first instance, as a model protein for molecular investigation. On the basis of the RSL of SsCEI, a set of four peptides, differing in size and nature at their P1 site, were designed and synthesized. Peptides SsCEI 1 and SsCEI 2 correspond to residues 119–134 and 123–134 of the SsCEI protein, respectively, and include the P1-P′1 (L126-E127) binding site which is reportedly involved in protease inhibition (Figure S1). The shorter variant (SsCEI 2), starting with the N-terminus of RSL, was designed to minimise peptide length while maintaining intact the RSL binding site. Two further peptides were projected, SsCEI 3 and SsCEI 4, to replace the P1 residue Leu with Ala, which is the preferred amino acid in the substrates of mammalian APEHs. The sequences of these peptides are reported in Table 1. Amidation at the C-terminal end was introduced to mimic the amino acid stretch within the protein backbone, whereas the amino termini of peptides were not acetylated to prevent substrate-like effects when in contact with APEH. Peptide structures within SsCEI protein inhibitor are predicted to be random/extended, as they have to be free in adopting the best conformation needed to dock the target proteases. Circular dichroism (CD) spectroscopy analyses were carried out to obtain information on the secondary structures of peptides outside the context of the native protein. Interestingly, the CD spectra measured between the 190 nm and 250 nm demonstrated that, except for SsCEI 4 which was largely unstructured (Figure S2), these peptides have well defined secondary structures in water. Specifically, CD spectra of SsCEI 2 and SsCEI 4 at 37°C, despite the single mutation, showed markedly different profiles, suggesting that the Leu→Ala substitution at the P1 site induces significant conformational alterations. CD spectra of SsCEI 2 featured canonical ‘α-helix’ curves with surprising fidelity (Figure S2). These data are in agreement with the role that the RSLs have in the native inhibitor proteins, and suggest a strong tendency of these peptides to adopt different conformations following even minimal sequence modifications.

**Table 1:** Synthetic peptides used in this study.

Peptide	Theoretical mass value (Da)	Measured mass value (Da±SD)	P1 P′1
SsCEI 1	1818.12	n.d.	YAIDTILL EIKNINAD
SsCEI 2	1355.61	1961±265	TILL EIKNINAD
SsCEI 3	1766.04	2012±150	YAIDTILA EIKNINAD
SsCEI 4	1313.53	1986±28	TILA EIKNINAD

Peptides were synthesized with a free amino group at the N-terminus and an amidic group at the C-terminus. The apparent MWs of SsCEI peptides were determined by gel filtration chromatography. The calibration curve (R2 = 0.995) was obtained using a set of synthetic peptides. Data reported are the result of three independent determinations.

CD spectra were also recorded in the temperature range between 37°C and 77°C, with increasing temperature steps of 10°C. Under these conditions, SsCEI 2 and SsCEI 3 showed considerable structural stability, as seen by the poor influence of temperature on their conformation (Figure S2). SsCEI 1 was not examined due to its poor stability in aqueous solution at the concentrations required for these analyses. These findings indicate that, in our model, the backbone architecture of the inhibitory loop is imposed by its specific amino acid sequence, and that the protein scaffold does not constrain the conformation of the RSL.

Given the relevant contents of β-sheet structures observed in SsCEI 2 and SsCEI 3, we next investigated the oligomerization properties of these peptides to exclude the occurrence of macroscopic aggregates. For this purpose, 100 µM solutions of peptides SsCEI 2, SsCEI 3 and SsCEI 4 were analysed by size-exclusion chromatography, and their apparent molecular masses were extrapolated from a calibration curve. As shown in Table 1, SsCEI 2, SsCEI 3 and SsCEI 4 were essentially monomers, suggesting that the secondary structures detected by CD were not a result of non-specific self-association, but seemed to be an intrinsic property of the peptides.

### APEHSs is specifically and efficiently inhibited by SsCEI 2 and t10c12-CLA isomer

**Figure 1:**
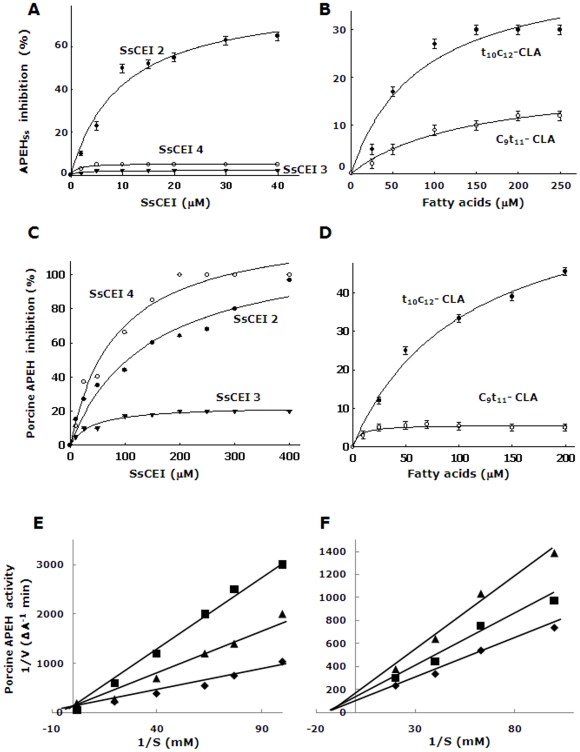
Kinetic analysis of SsCEIs and CLA isomers towards APEHs. Binding of increasing concentrations of the SsCEI peptides (A), and CLA isomers (B) to APEH_Ss_. Binding of SsCEI peptides (C), and t_10_c_12_-CLA (D) to porcine APEH. The hyperbolic curves indicate the best fits for the data obtained, with IC_50_ values calculated from the graphs. Inhibition kinetics analyses with porcine APEH (0.5 nM) at increasing SsCEI 4 concentrations: 100 µM (triangles) and 150 µM (squares) (E). Similarly, inhibition kinetics by increasing t_10_c_12_-CLA concentrations: 50 µM (squares) and 100 µM (triangles) (F). Enzyme incubated without inhibitors were used as control (diamonds) (E, F). The inhibition constants, Ki, were determined by the Lineweaver–Burk equation for competitive and non-competitive inhibition, respectively.

Preliminary experiments were aimed at investigating the possible interaction/inhibition between APEHSs and the peptides SsCEI 2, SsCEI 3 and SsCEI 4. Inhibition analyses were performed by pre-incubating the enzyme with increasing amounts of these compounds and their half-maximal inhibitory concentrations (IC50) were determined. The calibration curve for SsCEI 2 followed a hyperbolic pattern with an IC50 value of 9.8±1.0 µM as calculated using Ac-Leu-pNA as reporter substrate; however, in the presence of SsCEI 3 and SsCEI 4 no detectable decrease in the APEHSs activity was observed (Figure 1A). Therefore, APEHSs interacts with and is inhibited only by SsCEI 2 which has a Leu on the P1 site, suggesting that the residue on this position has a major role in the recognition with the target protease. Moreover, among the fatty acids tested, only the t10c12-CLA isomer was able to dose-dependently reduce APEHSs activity with an IC50 value of 80±2.0 µM, whilst no significant modulatory effect was observed using c9t11-CLA isomer (Figure 1B). Notably, APEHSs activity followed a Michaelis–Menten kinetic, both in the absence and in the presence of SsCEI 2 but only the Michaelis constant (Km) was affected by increasing concentrations of substrate, suggesting that SsCEI 2 behaved as a competitive inhibitor. It was confirmed by plotting the data according to the Lineweaver–Burk equation (data not shown), which allowed the calculation of a Ki value for SsCEI-2 of 1.00±0.02 µM. In contrast, in the presence of increasing amounts of t10c12-CLA isomer, only the Vmax of APEHSs was affected, indicating a non-competitive inhibition mechanism for t10c12-CLA, with a Ki value of 140±20 µM.

### Mammalian APEHs are inhibited by SsCEI peptides and t10c12-CLA

The inhibition activity of SsCEI peptides was also assessed using mammalian APEHs purified from both porcine liver and human colon carcinoma intestinal cells (Caco-2). These two enzymes share more than 90% sequence identity, as calculated by the ClustalW algorithm (http://www.ebi.ac.uk/Tools/msa/clustalw2/). Ac-Ala–pNA was again used as the preferential substrate for these mammalian APEHs. As shown in Figure 1C, both SsCEI 2 and SsCEI 4 dose-dependently decreased porcine APEH activity, although to different extents (IC50 values were 142±30 µM and 84±16 µM, respectively). Comparable IC50 values were obtained using human APEH (data not shown). Moreover, the affinity of SsCEI 4 towards porcine APEH was revealed by a Ki value of 4.0±0.8 µM, as determined by the Lineweaver-Burk plot, which also showed that SsCEI 4 is a competitive inhibitor of this enzyme (Figure 1E). The greater efficacy of SsCEI 4 over SsCEI 2, can be ascribed to the preference for an Ala residue, with respect to leucine, at the P1 site, assuming that the SsCEI–APEH association occurs in a substrate-like manner. Data also suggested that the additional N-terminal residues in SsCEI 3 (Table 1), negatively affected the inhibition capacity towards both APEHSs and mammalian APEH (Figure 1A, C).

Next, the modulatory effects of CLA isomers on porcine APEH were investigated. The dose-dependent reduction of enzyme activity followed a hyperbolic pattern in the presence of t10c12-CLA isomer with an IC50 value of 105±23 µM (Figure 1D), whilst c9t11-CLA was ineffective. In addition the Ki of t10c12-CLA towards porcine APEH was 140±20 µM and the Lineweaver-Burk plot revealed a non–competitive inhibition mechanism. This is the first evidence of a direct inhibition of APEH by a CLA isomer.

### SsCEI 4 and t10c12-CLA are selective, time-dependent and non covalent APEH inhibitors

The selectivity of SsCEI 2 and SsCEI 4 for porcine APEH (hereafter APEH) were analysed in biochemical assays using a panel of eukaryotic serine proteases comprising trypsin, α-chymotrypsin, elastase, carboxypeptidase Y, subtilisin and thrombin. Results showed that SsCEI 4 has no detectable effects on the proteases tested. In contrast, SsCEI 2 displayed an inhibition activity towards bovine α-chymotrypsin. The inhibition curve also followed a hyperbolic pattern with increasing SsCEI 2 concentrations, and gave an IC50 value of 21.9±6.4 µM (Figure S3). To further examine the SsCEI 4 specificity towards APEH, its inhibition activity was determined in a reaction mixture containing the entire set of proteases reported above. Under these conditions the inhibition efficiency and the Ki of SsCEI 4 towards APEH were comparable to those measured in the presence of the APEH alone (data not shown).

To investigate the molecular inhibition mechanisms of SsCEI 4 and t10c12-CLA, time-dependent experiments were carried out. Of note, SsCEI 4 and t10c12-CLA inhibited APEH (100% and 43%, respectively) only after pre-incubation with the enzyme for at least 20 min, suggesting that they behave as time-dependent inhibitors. Moreover, to exclude the formation of adducts or degradation products between SsCEI 4 and its protease target APEH, we analysed the incubation mixtures by reverse-phase HPLC chromatography. The lack of new peaks in the HPLC chromatogram and invariability of peak area suggested that neither peptide degradation nor covalent binding with APEH occurred under the assay conditions (Figure S3). Data thus indicated that SsCEI 4 is a highly selective, time-dependent and non covalent inhibitor of APEH.

### Proteasomal degradation of the cystic fibrosis transmembrane conductance regulator (CFTR) mutated protein is prevented by SsCEI 4 and t10c12-CLA

The enzymatic stability of SsCEI 4 in 10% FBS was evaluated as previously reported [19] and the peptide was completely stable for at least one week under the assay conditions (data not shown).

In light of a recently proposed cooperative role for the APEH–proteasome system in the control of protein turnover [15], we hypothesised that APEH could be used as a target to indirectly control/modulate proteasome functions. To support this idea, we conducted in vitro experiments using the selected APEH inhibitors (SsCEI 4 or t10c12-CLA) on the Baby Hamster Kidney (BHK) cell line stably expressing a mutant protein of the cystic fibrosis transmembrane conductance regulator (CFTR), known as ΔF508 CFTR-3HA (hereafter called CFTR-M), bearing the deletion of Phe508, one of the most common modification in patients with cystic fibrosis. Many of the mutations in the CFTR gene that cause cystic fibrosis interfere with the folding and biosynthetic processing of CFTR molecules in the endoplasmic reticulum. Specifically, some mutations, including the common ΔF508, decrease the efficiency of CFTR folding, reduce the probability of its dissociation from molecular chaperones, and largely prevent its maturation through the secretory pathway to the plasma membrane. These mutant CFTR molecules are rapidly targeted for proteolysis via the UPS [20]–[22].

**Figure 2:**
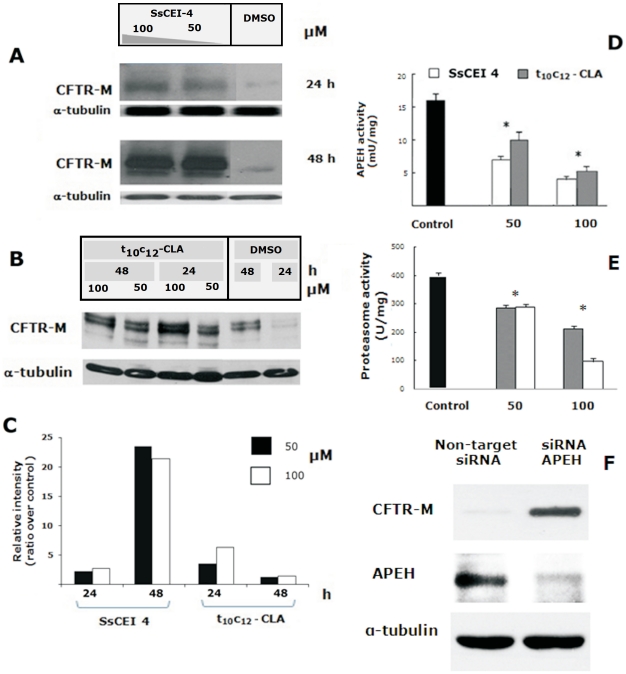
Analysis of the CFTR-M protein accumulation in BHK cells treated with the SsCEI 4 and t_10_c_12_-CLA and in APEH siRNA transfected CFBE41o-DF cells. Representative Immunoblots and associated densitometric analysis for cytosolic CFTR-M accumulation in BHK cells following 24 h and 48 h exposure to 50 µM or 100 µM SsCEI 4 (A), and to 50 µM or 100 µM t_10_c_12_-CLA (B). Bands were quantified using densitometric analysis and normalized against α-tubulin. The values were expressed as average fold increase as compared to untreated culture (C). APEH activity was measured in BHK cells incubated with 50 µM and 100 µM SsCEI 4 (white bars) or t_10_c_12_-CLA (grey bars) for 48 h (D). CT-like proteasome activities were measured in BHK cells incubated with 50 µM and 100 µM SsCEI 4 (white bars) or t10c12-CLA (gray bars) for 48 h (E). Untreated cultures were used as controls (black bars); the data are expressed as means±SD. *Significantly different (P<0.005) from the control (D, E). Representative Immunoblots of APEH and CFTR-M accumulation in CFBE41o-DF cells transfected with APEH siRNA. A scrambled, non-targeted siRNA, was used as negative control and α-tubulin was used as loading control (F).

Accordingly, BHK and human bronchial epithelial cells (CFBE41o-DF) expressing CFTR-M were used in this study as a model system to confirm the role of APEH in the coordinated protein-degradation machinery, and steady-state levels of the core-glycosylated CFTR-M form (140 kDa) were evaluated by immunoblot analysis. Remarkably, the SsCEI 4 peptide and the t10c12-CLA isomer efficiently prevented degradation of CFTR-M at the time intervals considered (24 h and 48 h). As a fact, exposure of BHK cells to 100 µM SsCEI 4 for 48 h or to 100 µM t10c12-CLA for 24 h induced a marked increase of CFTR-M levels (twenty- and five-fold, respectively) (Figure 2A, B, C), without any cytotoxic effects (data not shown). In addition, a dose-dependent inhibition of APEH and proteasome CT-like activities was observed upon 48 h of incubation (similar data on APEH and proteasome activities were measured after 24 h cell exposure) with both compounds, as shown in Figure 2D, E consistent with the immunoblot results.

Finally, siRNA technique was used to directly correlate APEH to the protein degradation processes via UPS. For this purpose, the accumulation of CFTR-M was evaluated in CFBE41o-DF cells following transfection with APEH siRNA. As shown in Figure 2F, APEH siRNA-transfected cells exhibited a considerable reduction of APEH protein levels and a marked accumulation of CFTR-M (eight-fold, data not shown) (Figure 2F), in contrast to cells transfected with a not specific siRNA which displayed basal levels of APEH and neglectable level of CFTR-M. Therefore, APEH can be seen as an alternative target, whose inhibition by competitive as well as non-competitive inhibitors is accompanied by a parallel down-regulation of proteasome activity through a yet unknown mechanism.

### SsCEI 4 and t10c12-CLA down-regulate APEH and proteasome activities in cancer cells

**Figure 3:**
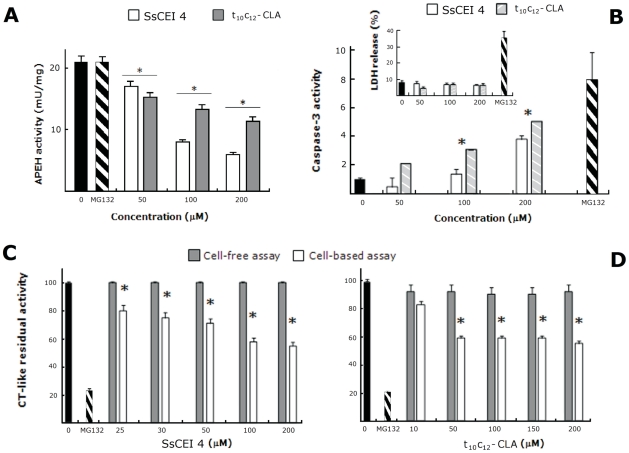
Down-regulation of the proteasome/APEH enzyme system by the SsCEI 4 and t_10_c_12_-CLA in Caco-2 cells. APEH activity was measured in Caco-2 cells incubated with 50 µM, 100 µM and 200 µM SsCEI 4 (white bars) or t_10_c_12_-CLA (gray bars) for 48 h (A). Proteasomal CT-like activity was measured in cell-free system (a partially purified proteasome fraction from differentiated Caco-2 cells, gray bars) and in Caco-2 cells (white bars) treated with increasing concentrations of SsCEI 4 (C) or t_10_c_12_-CLA (D). Caspase-3 activities and LDH release were measured upon 48 h incubations of Caco-2 cells with increasing concentrations of SsCEI 4 (white bars) and t_10_c_12_-CLA (striped grey bars) (B). The cytotoxic effect of the different treatments was evaluated by measuring the LDH release in the culture media (B insert). Cell-free protein mixtures, or Caco-2 cell cultures, treated with DMSO alone (black bars) or with MG132 (10 µM) (striped black bars) were used as positive controls. The data are expressed as means±SD. *Significantly different (P<0.005) from respective controls.

Proteasome inhibition represents a validated, although challenging, anticancer approach. However, to prevent the adverse effects deriving from indiscriminate cell death, inhibition of the proteasome needs to be tightly controlled or selectively induced in cancer tissues. Therefore, the concept that proteasome activity could be decreased via APEH inhibition was investigated in a cancer cell line. To this end, differentiated human colon carcinoma Caco-2 cells were treated with SsCEI 4, t10c12-CLA or with a specific PI (MG132) for 48 h. As shown in Figure 3A, SsCEI 4 and t10c12-CLA markedly reduced APEH activity in a dose-dependent manner, reaching their maximum effect at 200 µM, where enzyme activity was decreased by 70% and 50%, respectively. Under the same conditions MG132 treatment had no detectable effects.

We next examined the inhibitory effects of SsCEI 4 and t10c12-CLA on the CT-like proteasomal activity in Caco-2 cells and in cell-free assays. In these latter experiments, partially purified proteasome fractions from Caco-2 cells were used instead of the commercially available 20S proteasome or immunoproteasome. Indeed, it has been reported that in neoplastic cell lines the CT-like proteasomal activity, as well as the sensitivity to different PIs, is greatly influenced by the highly variable proteasome subunit composition [23].

Therefore, cell exposure to SsCEI 4 and t10c12-CLA, produced a dose-dependent decrease (up to 45% of the residual activity) of the CT-like proteasome activity with respect to the untreated cultures, whereas partially purified proteasome was not affected by these compounds (Figure 3C, D), thus confirming that it is not directly targeted by these inhibitors.

Next, we evaluated the effects of SsCEI 4 and t10c12-CLA treatment on the activation of caspases. As shown in Figure 3B, caspase-3 activity, a key effector of apoptosis, was improved at increasing doses of either SsCEI 4 or t10c12-CLA. This was not associated with any cytotoxic effect even at the highest concentration (200 µM), as indicated by the lactate dehydrogenase (LDH) activity levels which remained comparable to those of controls (Figure 3B insert).

Therefore, our results, consistently with the reporting coordinated functions of proteasome and APEH in protein turnover [15], add the relevant information that proteasome modulation could occur via a complex pathway which has APEH like an important and regulative factor. Moreover, since APEH activity is not influenced by cell treatment with the specific PI MG132 (Figure 3A), proteasome modulation should be hierarchically down-stream of APEH inhibition. This view is also corroborated by the observation that APEH and proteasome seem to have no direct interactions, as they are distinctly eluted from gel filtration columns loaded with protein extracts obtained from SsCEI 4-treated or untreated Caco-2 cells (Figure S4). Further investigations aimed at a better understanding of the molecular mechanism underlying the proteasome inhibition by APEH are currently in progress.

### The combined use of SsCEI 4 and t_10_c_12_-CLA improves the inhibition of proteasome activity, triggers apoptosis and increases the level of UPS substrates in Caco-2 cells

To finally confirm the reliability of the APEH-mediated strategy to affect UPS activity, several readouts were evaluated in differentiated Caco-2 cells treated with SsCEI 4 and t10c12-CLA, alone or in combination. Caco-2 cells were incubated for 48 h with SsCEI 4, t10c12-CLA (200 µM), or an equimolar mixture (100 µM each) of both compounds. The commercially inhibitors of APEH (ebelactone) or the proteasome (MG132) were used as positive controls.

**Figure 4:**
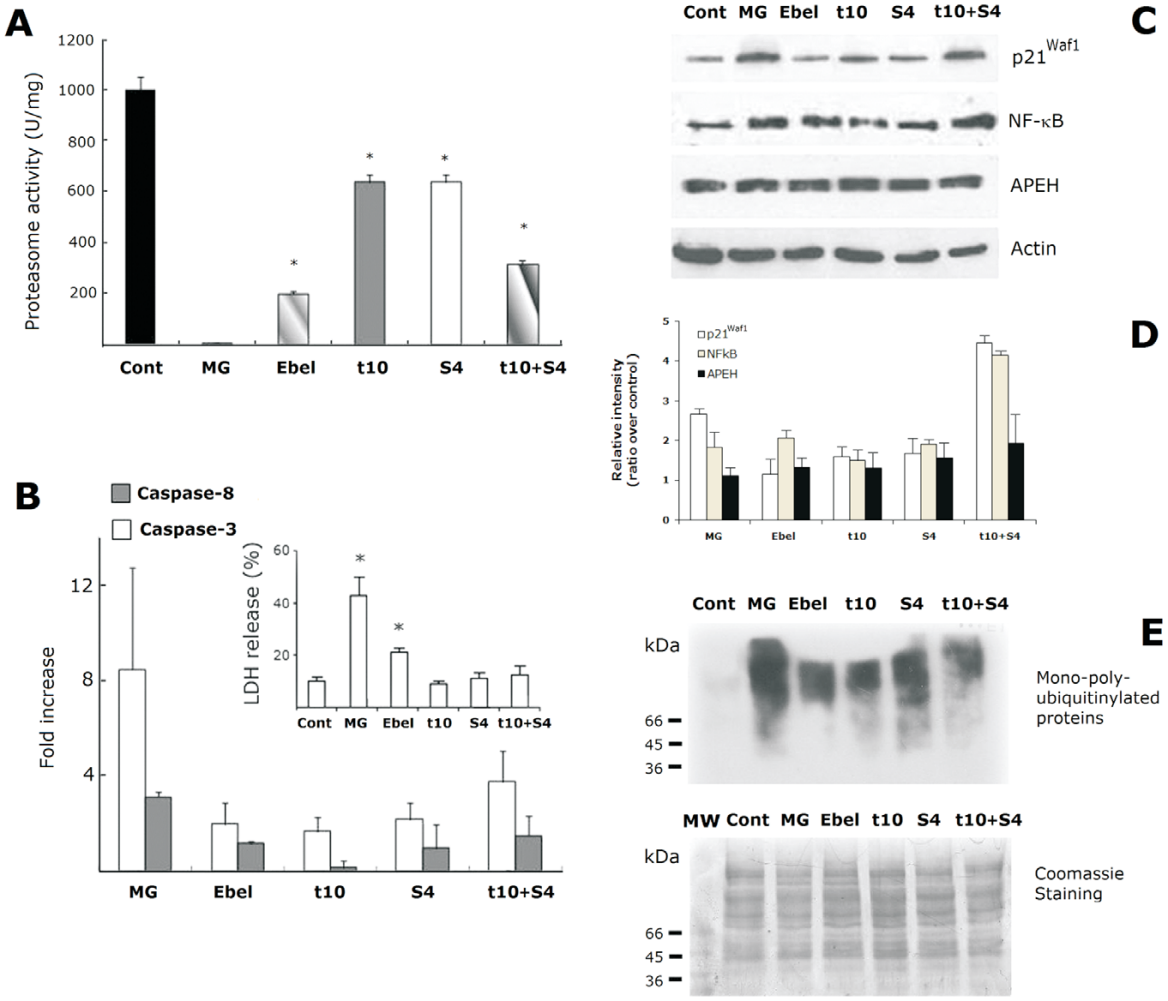
Evaluation of proteasome inhibition markers in Caco-2 cells incubated with SsCEI-4 and t_10_c_12_-CLA, alone or in combination. Caco-2 cells were treated (48 h) with 10 µM MG132 (MG), 100 µM ebelactone (Ebel), 200 µM SsCEI 4 (S4), t_10_c_12_-CLA (t10), or with a mixture of both (t10+S4). Cells exposed to DMSO alone were used as the controls (black bar). The data are expressed as means±SD. *Significantly different (*P*<0.005) from the control (A). Caspase-3 (white bars) and caspase-8 (grey bars) activities measured as fold increase in comparison to untreated cells (B). The cytotoxic effect of the different treatments was evaluated by measuring the LDH release in the culture media (B insert). Representative immunoblots of the expression of p21Waf1, NF-κB, and APEH in Caco-2 cell exposed for 48 h to MG, Ebel, S4, t10 or with a mixture of both (t10+S4) (C). Data on Western blot analysis are expressed as the density ratio of target to control (β-actin) in arbitrary units. The values were expressed as average relative intensity as compared to untreated cultures and expressed as means±SD of measurements performed in triplicate (D). Protein ubiquitinylation in Caco-2 cell exposed for 48 h to MG, Ebel, S4, t10 or with a mixture of both (t10+S4) (E, upper panel). Upon the immunodetection, the membrane was stained with Coomassie blue. The lane loaded with molecular mass markers [MW kDa] was shown (lower panel).

Cell exposure to SsCEI 4 or t10c12-CLA saw 40% reduction in the proteasomal CT-like activity, with a more marked decrease (about 73%) resulting from their combined use (Figure 4A). A similar behaviour was observed when APEH activity was monitored (data not shown), which supports the hypothesis of an additive effect (combination index; CI = 1) [24] produced by SsCEI 4 and t10c12-CLA on the target protease. However, due to the difficulties in the setting-up the large number of variables which possibly affect the formation of enzyme–inhibitor-substrate complexes, we were unable to reproduce the additive effects on the proteasome or APEH activities in cell-free assays.

To evaluate the pro-apoptotic effects arising from cell exposure to a mixture of SsCEI 4 and t10c12-CLA, we measured caspase-3 and caspase-8 activities as these enzymes have been reported to be essential for the proteasome-induced apoptosis cascade [6], [25]. Specifically, caspase-3 was significantly increased by SsCEI 4 or t10c12-CLA (about two-fold in both cases; P<0.01), with a further improvement produced by their combination (about four-fold), in comparison to untreated cultures. Although caspase-8 activity was less intense, it showed a profile similar to that of caspase-3, further supporting the view that cell death occurs by an apoptotic mechanism (Figure 4B). These data are in agreement with the well-established association between proteasomal inhibition and apoptosis induction, and confirm the additive effects produced by using a mixture of SsCEI 4 and t10c12-CLA on the caspase cascade in cancer cells. Notably, the toxicity resulting from the APEH-mediated inhibition of proteasome activity, as indicated by the treatment with SsCEI 4, t10c12-CLA or ebelactone, was significantly lower than that observed in culture incubated with MG132 (Figure 4B insert).

Moreover, the immunoblot analysis, showing that the levels of APEH in cells were not affected by any of these treatments, clearly indicated that the APEH down-regulation resulted from an enzyme inhibition process, rather than a reduction in protein expression. Cell exposure to SsCEI 4 and t10c12-CLA, alone or in combination, produced an increase of well-known proteasome substrates (p21Waf1 and NF-κB, two-fold or four-fold, respectively; Figure 4C, D). These findings are consistent with the idea that the relationship between apoptosis and the accumulation of damaged or short-lived regulatory proteins has a prominent role in controlling the homeostasis of cancer cells [22]. Cytoplasmic increase of NF-κB levels is indeed regarded as a major hallmark of apoptotic cells, since NF-κB nuclear translocation, following I-κB degradation by UPS and gene transcription, is a well-established mechanism of cell growth. Proteasome inhibition in cancer cells leads to a reduced rate of I-κB degradation, and to a longer persistence of NF-κB in the cytoplasm [26], [27]. In the same way, accumulation of p21Waf1, a negative regulator of the cell division cycle, is a direct evidence of increased apoptosis and of reduced proteasome activity, since it has been reported that its degradation occurs through N-terminal as well as internal lysine ubiquitinylation [28].

Polyubiquitinylated proteins are normally degraded by the cellular proteasomes, and down-regulation of proteasome activity has been shown to substantially suppress bulk intracellular protein turnover [29]. As evidenced in Figure 4E, following incubation with SsCEI 4, t10c12-CLA (alone or in combination), ebelactone or MG132, we detected in cell extract the presence of high-molecular-mass immunoreactive species (66 kDa to 160 kDa) which are absent in untreated cultures. These signals are indicative of polyubiquitin conjugates in the treated cells, confirming that these compounds deregulate UPS activity in cancer cells.

As a whole, our in vitro results demonstrate that APEH inhibition by SsCEI 4 and t10c12-CLA treatments is associated with increased levels of the typical markers of proteasome inhibition without any cytotoxic effect. Although pro-apoptotic activities of CLA mixtures and their ability to induce an increase of the cytoplasm levels of NF-κB and p21Waf1 have been previously reported [30], [31], to our knowledge this is the first study showing that their effects are mediated, at least in part, by the APEH/proteasome system, suggesting a possible mechanism by which CLA isomers exert their anticancer activity.

### Putative APEH binding site for t_10_cis_12_-CLA isomer

In light of these outcomes, APEH inhibition represents a novel strategy to regulate proteasome activity, with potential applications in biomedical fields. Knowledge of the enzyme–inhibitor binding sites at the molecular level is pivotal for our understanding of the underlying mechanisms, as well as for the design of novel and more efficient inhibitors. In a previous work [17], the structural model of the inhibition complex APEHSs-SsCEI protein corroborated by mutagenesis studies, indicated an involvement of the SsCEI RSL in the interaction with the active site of the enzyme target. Therefore, it is conceivable to assume that the competitive inhibition of APEHSs by SsCEI 4 peptide occurs through a similar binding-mode.

The surprising down-regulation of APEH by t10c12-CLA and the finding of additional non-competitive APEH binding pockets apart from the active site, prompted us to undertake a molecular modelling study to look for potential APEH-CLA binding sites. Protein-fatty acid docking analyses were carried out starting from the previously reported structural model of APEHSs [17], herein used for the biochemical investigation. The APEHSs 3D model, built on the X-ray structure of APEH from Aeropyrum pernix [32], shows the typical features of a POP family member: a α/β-hydrolase catalytic domain with the (Ser-Asp-His) catalytic triad, covered by a central tunnel of an unusual β-propeller domain.

**Figure 5:**
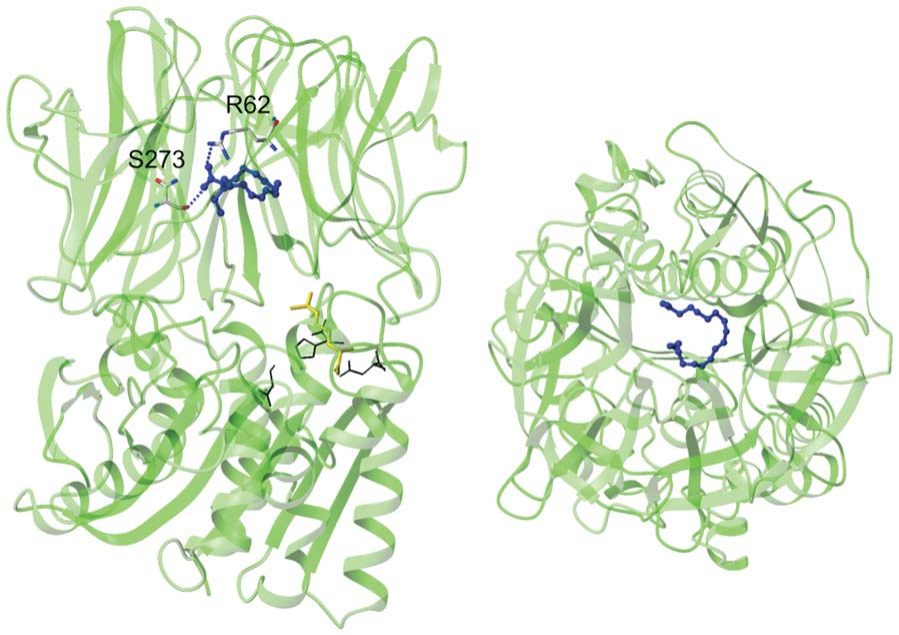
Binding mode of the t_10_c_12_-CLA with APEH_Ss_. Binding mode suggested by docking analysis for t_10_c_12_-CLA (blue; ball-and-stick mode) with APEH_Ss_ (cartoon representation; green, left). Protein residues involved in stabilising the interactions with the carboxylic group of the t_10_c_12_-CLA are represented as sticks. The Ser-Asp-His catalytic triad residues are shown as black lines; R507 is shown in yellow. (right) View rotated 90° along the x-axis (the horizontal axis parallel to the image plane).

**Figure 6:**
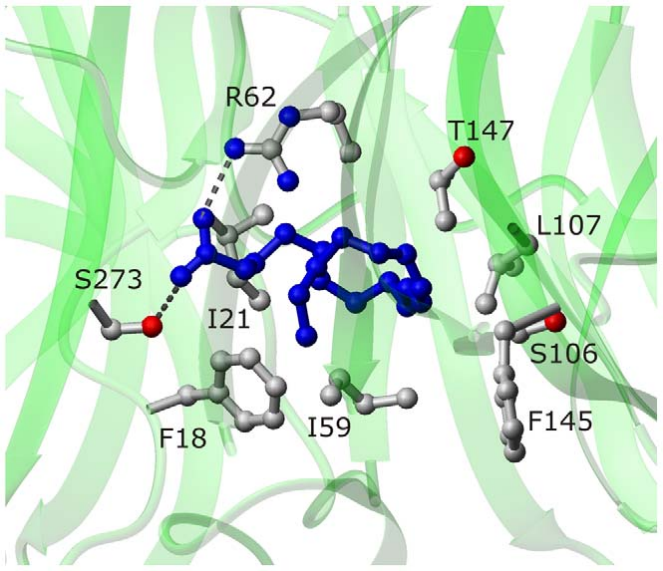
Suggested binding site on APEH_Ss_.by docking analysis for the t_10_c_12_-CLA (blue; ball-and-stick mode) isomer. The relevant APEH_Ss_ residues are shown in ball-and-stick representation.

Docking calculations were performed by using the AutoDock simulation package [33]. The docked conformations of t10c12-CLA suggested two putative binding modes that were characterised by different anchoring points for the carboxylate group of the CLA isomer: the positively charged side-chains of either R62 or R507. However, the binding involving the residue R507 appeared to be in conflict with the non-competitive inhibition mechanism indicated by the experimental data, as R507 belongs to the active site of APEHSs [17]. Thus we did not consider this binding approach further. binding of t10c12-CLA involving R62 residue of the enzyme is in agreement with the non-competitive mechanism of inhibition resulting from cell-free assays. In this case, t10c12-CLA occupies the β-propeller tunnel, eventually obstructing the passage of the substrate and/or the product (Figure 5). Details of the binding mode involving R62 are shown in Figure 6: t10c12-CLA carboxylate group interacts with the side-chains of R62 and S273 of APEHSs, while the long hydrophobic carbon tail of t10c12-CLA is stabilised by van der Waals interactions with some of the hydrophobic residues that line the β-propeller tunnel of APEHSs.

Of note, the interaction mechanism suggested by this docking analysis shares common characteristics with the fatty-acid-binding proteins (FABP) [34], [35]. X-ray structural studies have shown that the fatty acid molecule binds to the relatively large FABP inner cavity, and is anchored to a positively-charged arginine residue and a polar amino acid (usually serine or threonin), with the hydrophobic tail again stabilised by van der Waals interactions with hydrophobic residues.

The lack of structural information and the difficulty to predict a sufficiently accurate 3D model for any mammalian APEH have prevented us from performing modelling studies on mammalian APEHs. However the functional properties indicate significant similarity between mammalian and archaeal APEHs, showing that both are inhibited by SsCEI 4 peptide in a competitive manner (Figure 1E) or by t10c12-CLA through a non-competitive mechanism (Figure 1F), with comparable Ki values. Therefore, we hypothesise that the enzymes from archaeal and mammalian sources could share some common features in their modes of interaction with these inhibitors suggesting that APEHSs can be used as an initial model system for the early design of novel inhibitors of mammalian APEH.

### Conclusions

Proteasome is an abundant multi-enzyme complex that provides the main pathway for the protein turnover or the elimination of misfolded and aggregated proteins. As such, it controls the levels of proteins involved in cell-cycle progression and apoptosis in normal and malignant cells, and has become an important therapeutic target in anticancer therapies. A large number of specific PI molecules have been developed to date [36], but despite their indisputable efficacies all of these suffer for negative side-effects. These events represent the major drawback of impairing the activity of a target largely involved in physiological processes. For these reasons, several studies have suggested that the targeting of functionally related, up-stream or down-stream proteasome effectors [29], can be an alternative and a safer way to recover proteasome dysfunction associated with pathological conditions [29], [37], [38].

In this study we showed for the first time that, by using a set of selected APEH inhibitors, proteasome activity can be regulated through an APEH-mediated mechanism which represents a novel strategy to control UPS functions. Beside these findings, we demonstrated that the stable, selective and non toxic inhibitors of APEH (a synthetic peptide and a CLA isomer) are able to produce a noticeable down-regulation of UPS activity in cells. Moreover, these molecules represent attractive templates for the design of more potent inhibitors, with potential applications as anticancer and anti-inflammatory agents. In addition, the synergistic effects resulting from their combined use strongly suggest that chimeric compounds, including competitive and non-competitive inhibitors, with increased specificity and enhanced activity, can be investigated and developed.

APEH has been postulated to serve as a key regulator of N-terminally acetylated proteins [39] but the biological effects of disrupting APEH has not been completely understood. As more than 80% of proteins in human cells are N-terminal acetylated [40]–[42] and protein acetylation is implicated in a variety of essential cellular pathways [43], it is thus likely feasible that APEH is involved in these processes.

As reported in previous studies, proteasome and APEH act cooperatively in protein turnover [15], [44], although the biochemical mechanisms remain to be clarified. In this regard, in contrast to the general idea that N-terminal acetylation protects from degradation, in certain proteins some sequences which include acetyl groups at the N-terminus were recently found to be involved in degradation signals [45], [46]. On the basis of our preliminary results, a direct interaction between APEH and proteasome might be excluded, whereas the hypothesis that APEH can activate or stabilise the proteasome by uncovering the N-tail of a yet unknown negative effector protein cannot be ruled out. Of note, we showed that whereas APEH inhibition triggered an impairment of the proteasome activity, its selective inhibition did not affect APEH functions, likely suggesting that APEH could be an up-stream modulator of the proteasome. Studies aimed at achieving a better understanding of the mechanism/s responsible for the APEH-mediated down-regulation of proteasome and at the evaluation of APEH inhibitors in animal cancer model are currently in progress.

## Materials and Methods

### Reagents

Pure c_9_t_11_- and t_10_c_12_-CLA isomers and caspase-3 and -8 fluorometric Assay Kits were purchased from Sigma-Aldrich. DMEM/F12, DMEM, L-glutamine, penicillin-streptomycin and FBS were from Gibco-BRL. Porcine liver APEH was obtained by Takara. Chemicals of the highest purity were from Sigma-Aldrich or Calbiochem.

### Peptide design, synthesis and characterisation

The peptides were prepared as amidated derivatives by solid-phase synthesis (synthesis scale, 0.1 mmoles), following standard Fmoc/tBu protocols [47]. A rink amide resin (substitution, 0.57 mmol/g) and amino acid derivatives with standard protection were used in all of the syntheses. Cleavage from the solid support was performed by treatment with a trifluoroacetic acid (TFA)/tri-isopropylsilane/water (90∶5∶5, v/v/v) mixture for 90 min at room temperature. The crude peptides were precipitated in cold ether, dissolved in a water/acetonitrile (1∶1, v/v) mixture and lyophilised. The peptides were purified by reverse-phase HPLC using a semi-preparative 5×1 cm ID C18 monolythic Onyx column, applying a linear gradient of 0.05% TFA in acetonitrile from 10% to 70% over 8 min at a flow rate of 15 µL/min. Peptide purity and identity were confirmed by liquid chromatography–mass spectrometry analysis.

### Gel filtration analysis of synthetic peptides

Gel filtration chromatography was performed on a BioSep SEC-S2000 column equilibrated with 50 mM phosphate buffer pH 6.8, at a flow rate of 1.0 mL/min. A standard curve was built using a set of synthetic peptides with molecular weights between 1.500 amu and 2.500 amu. For this purpose, peptide aliquots were injected onto the column and a plot of KD versus log10 molecular weights (MW) was obtained, where KD = (Ve-Vo)/(VT-Vo), Ve is the elution volume of the sample, and VT and Vo are the total and void volumes of the column, respectively.

### Circular dichroism spectroscopy

CD spectra were obtained on a Jasco J-715 spectropolarimeter with 400 µL of 8.0×10-7 M protein in 5 mM Tris–HCl pH 7.5. Hellman quartz cells of 0.1-cm-path length were used in the far UV (190–250 nm). The temperature of the sample cell was regulated by a PTC-348 WI thermostat and thermal CD was performed from 250 to 195 nm by raising the cell temperature from 37°C to 77°C. The thermal CD spectra were signal-averaged by combining three scans and the baseline was corrected by subtracting a buffer spectrum. The samples were then cooled back to 37°C to monitor the final folding of the peptides.

### Purification of APEHs and proteasome

APEH from Sulfolobus solfataricus was purified as previously reported [17]. Partial purification of human APEH and proteasome was carried out from protein extracts of Caco-2 cell. Briefly, protein extracts (500 µg) were fractionated by gel filtration chromatography on a Superdex 200 PC 3.2/30 column connected to a SMART System (Pharmacia) equilibrated in buffer 50 mM Tris-HCl, pH 7.5, 100 mM NaCl, at 0.1 mL/min. The eluted fractions were assayed using the specific substrates for APEH and the proteasome. The active fractions were collected and used for further analysis.

### Enzyme assays

Porcine liver APEH activity was measured spectrophotometrically using the chromogenic substrate acetyl-Ala-pNA (Bachem). The reaction mixture (1 mL) containing pure APEH (38 ng) or an appropriate amount of cell extract in 50 mM Tris-HCl buffer pH 7.5 (Tris Buffer), was preincubated at 37°C for 2 min. Then, 1 mM acetyl-Ala-pNA was added and the release of p-nitroanilide (ε410 = 8800 M-1 cm-1) was measured by recording the absorbance increase at 410 nm on a Cary 100 Scan (Varian) UV/Vis spectrophotometer, equipped with a thermostated cuvette compartment. APEH activity was expressed in IU. The APEHSs activity was measured using acetyl-Leu-pNA (0.1 mM) (Sigma) as substrate. The reaction mixture (1 mL) containing the appropriate amount of enzyme in 25 mM Tris-HCl buffer pH 7.5, was preincubated at 80°C for 2 min. Then, 0.1 mM acetyl-Leu-pNA was added and the release of p-nitroanilide was measured, following the standard assay procedure described above.

The synthetic fluorescent substrate N-succinyl-Leu-Leu-Val-Tyr-7-amido-4-methylcoumarin (N-Suc-LLVT-AMC) was used for the measurement of the CT-like activity of the proteasome, at a final concentration of 0.080 mM. The reaction mixture (0.9 mL) containing appropriate amount of proteasome was preincubated as above, in Tris buffer. N-Suc-LLVT-AMC was added, and the release of the fluorescent product (7-AMC) was monitored for 5 min in a Perkin–Elmer LS 50B fluorimeter. The excitation and emission wavelengths were 380 nm and 460 nm, respectively.

The carboxypeptidase Y, elastase, thrombin, trypsin and subtilisin activities were also evaluated according to previously published methods [18].

### Enzyme inhibitory assays

Protease inhibitor activities of the SsCEI peptides and the CLA isomers were carried out using a fixed amount of APEH or partially purified proteasome (3–5 nM and 0.12 mg/mL, respectively), and increasing the SsCEI and CLA isomer concentrations. Mixtures were pre-incubated for 30 min at 37°C before the addition of the substrate, and the enzymatic activities were followed as described above. Protease inhibitor activities of the SsCEI peptides and the CLA isomers were determined towards APEHSs. The protease and increasing concentrations of the inhibitors were mixed and preincubated for 30 min at 80°C before the addition of the specific substrate. The residual enzymatic activity was determined using the assay procedure described above.

The time-dependent inhibition of SsCEI 4 and t10c12-CLA towards APEH was assessed. Mixtures containing appropriate amounts of each inhibitor and of APEH were pre-incubated for 20 min at 37°C; they were then diluted (1∶5) into the standard assay mixture, which contained the substrate only. The enzymatic activity was followed as described above. Control samples were prepared by pre-incubating the same amounts of APEH without the inhibitors and then diluted in the standard assay mixture.

The additive effects elicited by cell exposure to SsCEI 4 and t10c12-CLA was calculated accordingly to the Chou and Talalay equation (CI = D1/[(DM)1×[fa/(1-fa)]1/m1+D2/[DM2×[fa/(1-fa)] 1/m2) [24].

### HPLC analysis of SsCEI peptides incubated with the target enzyme

The experiments were conducted at 37°C in 50 mM Tris buffer, pH 7.5. Solutions of SsCEI 4 and porcine APEH were incubated for up to 2 h at optimal concentrations (4 µM SsCEI 4 and 100 µM APEH) to guarantee a high degree of enzymatic inhibition. At specific time intervals, 195 µL aliquots were taken, and the reaction was stopped by addition of 5 µL TFA. The samples were then analysed directly by reverse-phase HPLC (Dionex BioLC) on a μBondapak C18 column (3.9×300 mm, Waters), eluted with a linear gradient (0–60% acetonitrile in 0.1% TFA) at a flow rate of 1 ml/min. Control peptide samples were incubated in the absence of the purified enzymes and run in parallel.

### Antibodies

The following antibodies were used: anti-APEH antibody (sc-102311; Santa Cruz Biotechnology); anti-NF-κB/p65 antibody (Thermo Scientific); anti-p21Waf1 antibody (Exbio); pan Ab-5 anti-actin antibody (clone ACTN05, Thermo Scientific); and monoclonal antibodies against polyubiquitinylated proteins conjugated with horseradish peroxidase (FK2H, Enzo, Life Science). The ΔF508CFTR-3HA protein was detected with an anti HA monoclonal antibody (Covance).

### Cell culture

BHK cells stably expressing CFTR-M (kindly donated by Dr. David Y Thomas, McGill University Montreal Canada) were cultured in DMEM/F12, 5% FBS, 1 mM L-glutamine, 200 µg/mL methotrexate, and 100 units/mL penicillin-streptomycin, at 37°C in a humidified 5% CO2 atmosphere. The cells were plated in 12-well plates, to a confluence of 60% for the 24-h incubations, and 40% for 48-h exposure to PIs. These treatments were initiated 24-h after the plating of the cells. Phase-contrast images of the cells were taken just before the lysis of the cells for protein analysis, using a Lica DM6000 inverted microscope.

Caco-2 cells (ATCC) were cultivated in DMEM supplemented with 10% FBS, 1 mM glutamine and 100 units/mL penicillin-streptomycin at 37°C in a humidified 5% CO2 atmosphere. The cells were studied between passages 12 and 22. The cells were split using trypsin-EDTA solution and plated in 6-well plates at a density of 8×104 cells/mL and the medium was replaced every 2–3 days. Under these conditions, the cells reached visual confluence after 7 days and the differentiated stage two weeks later. The differentiated cells were incubated for 48 h with the different substances.

### Protein extraction and Western blotting analysis

Following the treatments, the BHK and Caco-2 cells were washed three times with ice cold phosphate-buffered saline and collected immediately at 4°C in lysis buffer (1% Triton X-100, 0.1% SDS, 20 mM Tris-HCl, pH 8.0, 150 mM NaCl, 0.5% sodium deoxycholate and complete protease inhibitors [Roche]). The lysates were centrifuged at 10,000×g for 15 min at 4°C. The protein concentrations in the clear supernatants were determined (BCA protein assay reagent kit; Pierce) before their use in enzymatic assays or SDS–PAGE. In brief, for Western blotting, aliquots were run on SDS-PAGE (8% or 12.5%) and then electroblotted onto nitrocellulose (Schleicher & Schuell) or PVDF membranes (Immobilon™, Millipore). The membranes were next incubated with primary antibodies and then with the appropriate dilution of secondary antibody (1 h at 37°C). At the end of this time, the immunocomplexes formed were visualised by enhanced chemiluminescence and autoradiography according to the manufacturer protocol (Amersham Biosciences) and quantified by densitometric analysis with ChemiDoc XRS (Bio-Rad). Protein expression data was quantified with Quantity One Software (Bio-Rad).

### Apoptosis assays

The pro-apoptotic ability of the peptides and the CLA isomers were assayed by measuring the caspase-3 and caspase-8 activities using fluorometric kits, according to the manufacturer instructions. These assays were based on hydrolysis of the substrate acetyl-Asp-Glu-Val-Asp-7-amido-4-methylcoumarin (Ac-DEVD-AMC) or acetyl-Ile-Glu-Thr-Asp-7-amino-4-methyl coumarin (Ac-IETD-AMC) by caspase-3 and caspase-8, respectively. The release of the 7-AMC moiety in protein extracts prepared from the differently treated cells was evaluated by fluorimetry (excitation 360 nm, emission 460 nm). Their amounts were calculated by means of a standard curve prepared with pure AMC, and following normalisation for protein content, the activities were expressed as nmoles AMC/mg protein/min.

### Cytotoxicity assay

The release of LDH was used as the marker for cell toxicity [48]. The culture supernatants were sampled at the end of the incubations and centrifuged (4,000×g, 5 min, and 4°C). Aliquots of the clear supernatant (10 µL) were incubated with 190 µL reaction buffer (200 mM Tris/HCl, pH 8.0, 0.7 mM p-iodonitrotetrazolium violet, 50 mM L-lactic acid, 0.3 mM phenazine methoxysulphate, 0.4 mM NAD,) for 30 min at 37°C. Absorbance was measured at 490 nm and the results were expressed as percentages of total LDH release from control cultures treated with 1% (w/v) Triton X-100 and calculated as: [(experimental value - blank value)/(total lysis -blank value)−100].

### Docking calculations

The AutoDock (version 4.0) programme package [33] was chosen to dock t10c12-CLA into the large inner cavity of the APEHSs enzyme. The previously reported atomic coordinates of the APEHSs model [17], were used in the calculations. Amber charges and polar hydrogens were added to the protein using the PDB2PQR server (http://pdb2pqr-1.wustl.edu/pdb2pqr/). The ligand coordinates were generated by the PRODRG server (http://davapc1.bioch.dundee.ac.uk/prodrg/), and subsequently energy minimised using the Insight II package; atom charges and active torsions were defined using AutoDockTools. Affinity grids with dimensions 80×80×90 points (with spacings of 0.375 Å) were centred approximately in the middle of the enzyme β-propeller central tunnel and were large enough to cover the entire inner cavity of the enzyme subunit. The Lamarckian genetic algorithm and the pseudo-Solis and Wets methods were used for the conformational search. The maximum number of energy evaluations was set to 2.5×107 and a maximum number of 2.7×105 genetic algorithm operations were generated on a single population of 150 individuals. The operator weights for crossover, mutation, and elitism were set as the default parameters: 0.80, 0.02, and 1.0, respectively. One hundred runs were performed. The resultant docked conformations of the ligand were clustered and ranked according to the default AutoDockTools scoring function using a RMSD deviation of 3.5 Å. The MOLMOL programme was used for the molecular visualisation and analysis [49].

### Small interfering RNA transfection

The siRNA is purchased from Sigma (siRNAID SASI_Hs01_00240856 and SASI_Hs01_00240857), and CFBE41o-DF espressing ΔF508 CFTR were kindly provided by Dr. J. P. Clancy Department of Pediatrics, the University of Alabama at Birmingham, Birmingham, AL, USA. CFBE41o-DF cells at 5×104 cells/well were cultured over-night on 12-well plates and transfected 24 h after with APEH siRNA at a final concentration of 50 nM. using Lipofectamine 2000, according to the manufacturer's instruction. Non-targeting siRNA was used as a negative control. After 72 hours of transfection, cells are lysed in RIPA buffer and protein levels were determined by western blotting

### Statistical analysis

All data were obtained from triplicate analyses of three different preparations. Data were presented as means ±S.D. Statistical analysis and IC50 values were calculated with the SigmaPlot 10.0 software through a non-linear curve-fitting method and using a simple binding isotherm equation. Groups were compared by Student's t test, and P<0.05 was considered as significant.

## Supporting Information

Figure S1 **Amino acid sequence of SsCEI protein.**
(PDF)

Figure S2 **Far-UV CD spectra of the SsCEI peptides at different temperatures.**
(PDF)

Figure S3 **Binding of increasing concentration of the SsCEI peptides (as indicated) to a-chymotrypsin.**
(PDF)

Figure S4 **Gel filtration chromatography on a Superdex 200 column of protein extract from Caco-2 cells treated with 200 µM of SsCEI 4.**
(PDF)
