# Supplementary material for: Acylpeptide Hydrolase Inhibition as Targeted Strategy to Induce Proteasomal Down-Regulation
Source: PLoS One. 2011 Oct 10;6(10):e25888. doi: 10.1371/journal.pone.0025888 (PMC3189933; doi:10.1371/journal.pone.0025888)
Supplement: Figure S1 — Amino acid sequence of SsCEI protein. (PDF) [file pone.0025888.s001.pdf]

A

SsCEI

10 20 30 40 50

MNSESIYSMRVVSSAFKNEDFIPIKYTCDGQDLSPELEWDLVTNAKSYAIIVEDPDAP

60 70 80 90 100 110

GGTFIHWVIYNITTNRLPEGVPRLYKSQYGVQGVNDFGNIGYNGPCPPKTHPPHRYYF

120 **130** 140 150

YV**YAIDTILLEIKNINAD**KLKSLMEGHGIERGFVMGKYKRK

SsCEI 2

SsCEI 1

B

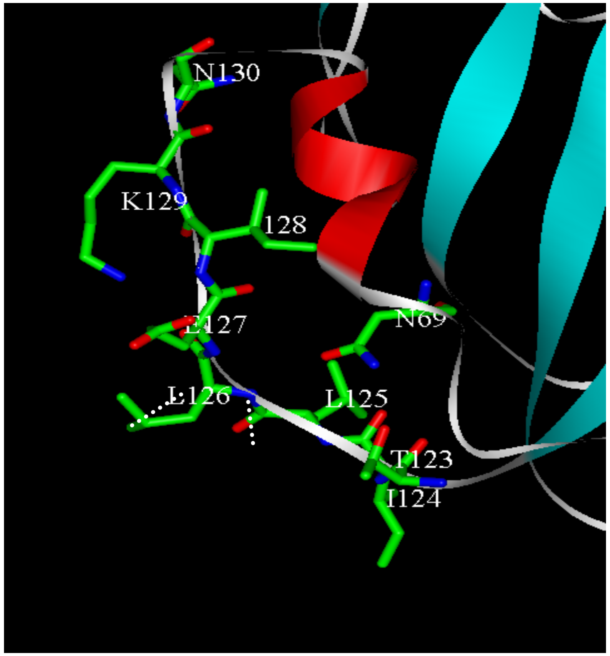

Figure S1: Amino acid sequence of SsCEI protein. The sequence of SsCEI 1 and SsCEI 2 are indicated in red and blue boxes, respectively (**A**). Model of the RSL of SsCEI (**B**).
