# Supplementary material for: Acylpeptide Hydrolase Inhibition as Targeted Strategy to Induce Proteasomal Down-Regulation
Source: PLoS One. 2011 Oct 10;6(10):e25888. doi: 10.1371/journal.pone.0025888 (PMC3189933; doi:10.1371/journal.pone.0025888)
Supplement: Figure S2 — Far-UV CD spectra of the SsCEI peptides at different temperatures. (PDF) [file pone.0025888.s002.pdf]

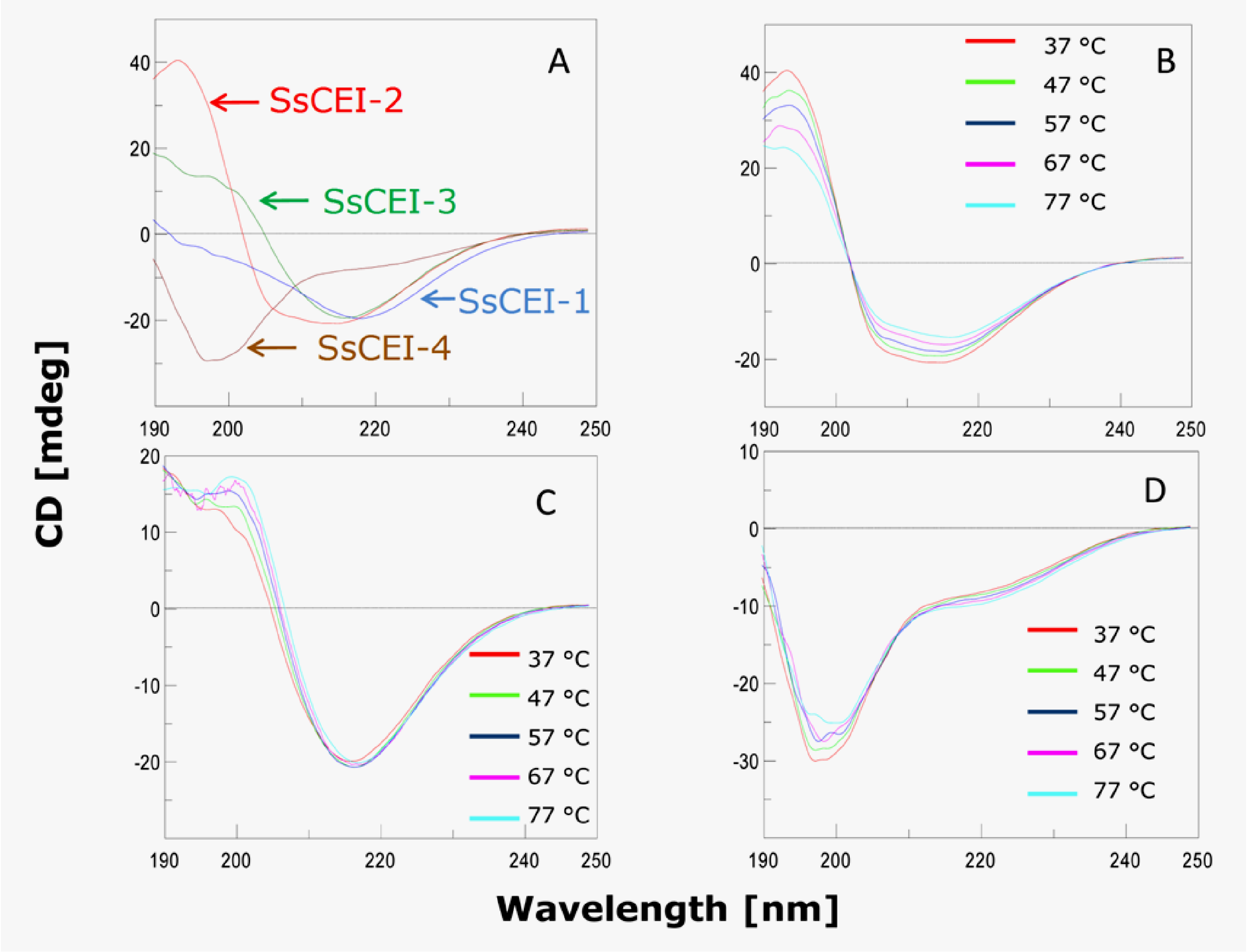

Figure S2: Far-UV CD spectra of the SsCEI peptides at different temperatures. SsCEI 1-4 at 37 °C (**A**), SsCEI 2(**B**), SsCEI 3 (**C**), SsCEI 4 (**D**), in the temperature range of 37 °C to 77 °C (as indicated). All the spectra were taken in water.
