# Supplementary material for: Acylpeptide Hydrolase Inhibition as Targeted Strategy to Induce Proteasomal Down-Regulation
Source: PLoS One. 2011 Oct 10;6(10):e25888. doi: 10.1371/journal.pone.0025888 (PMC3189933; doi:10.1371/journal.pone.0025888)
Supplement: Figure S3 — Binding of increasing concentration of the SsCEI peptides (as indicated) to a-chymotrypsin. (PDF) [file pone.0025888.s003.pdf]

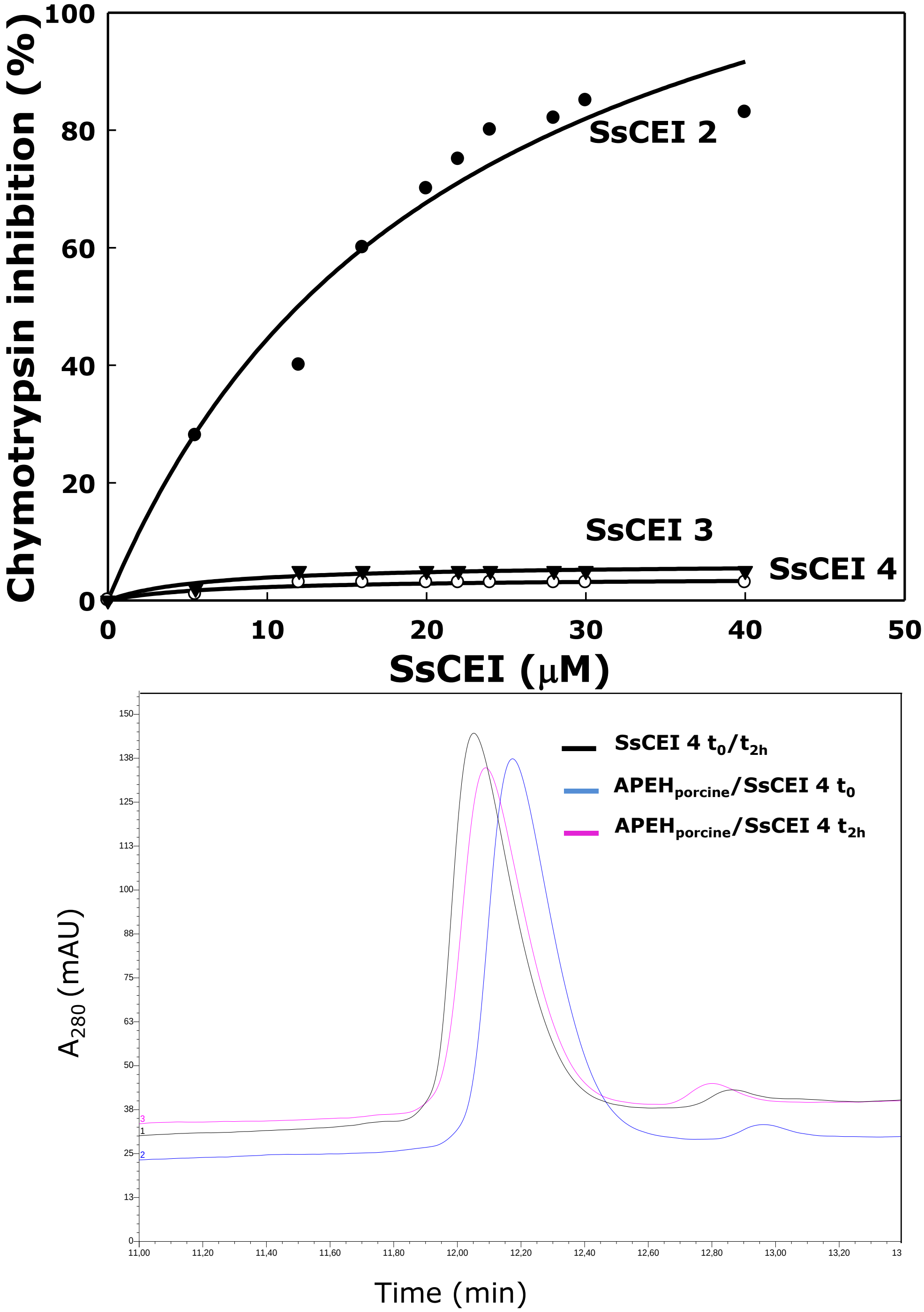

Figure S3: Binding of increasing concentration of the SsCEI peptides (as indicated) to α-chymotrypsin. The hyperbolic curve indicates the best fits for the data obtained, with IC_{50} value calculated from the graphs (upper panel). Representative chromatogram from RP C18 column analysis of SsCEI 4 incubated without or with porcine APEH, for different times (as indicated) (lower panel).
