# Supplementary material for: Acylpeptide Hydrolase Inhibition as Targeted Strategy to Induce Proteasomal Down-Regulation
Source: PLoS One. 2011 Oct 10;6(10):e25888. doi: 10.1371/journal.pone.0025888 (PMC3189933; doi:10.1371/journal.pone.0025888)
Supplement: Figure S4 — Gel filtration chromatography on a Superdex 200 column of protein extract from Caco-2 cells treated with 200 µM of SsCEI 4. (PDF) [file pone.0025888.s004.pdf]

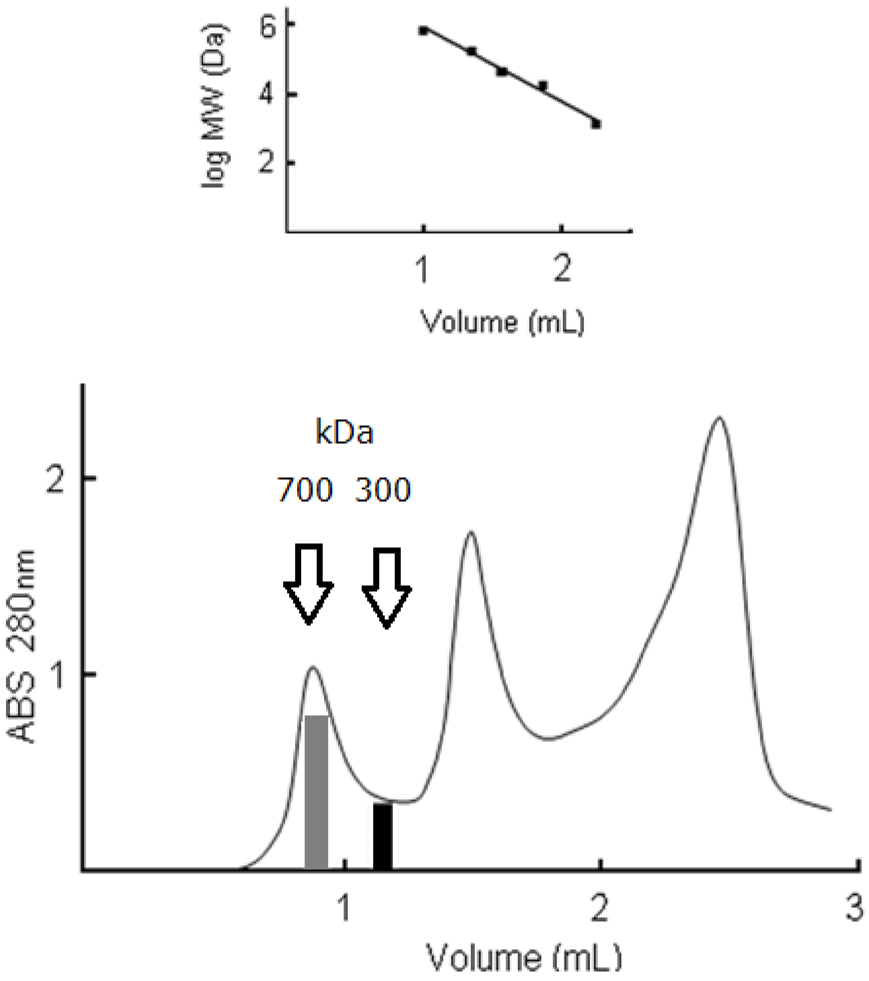

Figure S4: Gel filtration chromatography on a Superdex 200 column of protein extract from Caco-2 cells treated with 200 μM of SsCEI 4. The Superdex 200 column was calibrated using a set of gel filtration protein standards, including thyroglobulin (670 kDa), bovine γ-globulin (158 kDa), chicken ovalbumin (44.0 kDa), equine myoglobin (17.0 kDa) and vitamin B_{12} (1.35 kDa). The bars and ind[illegible]ate th[illegible] enzymatic activities of the proteasome (700 kDa) and APEH (300 kDa).
